# Informed consent for whole genome sequencing in mainstream clinics: logistical constraints and possible solutions

**DOI:** 10.1038/s41431-023-01520-8

**Published:** 2024-01-04

**Authors:** Amina Chaouch, Fiona Ulph, James Alder, Hisham Hamdalla, John Ealing, Tara Clancy, Rhona Macleod, Angus John Clarke

**Affiliations:** 1 Manchester Centre for Clinical Neurosciences, Salford Royal NHS Foundation Trust, Manchester, M8 6HD UK; 2 University of Manchester, Manchester, UK; 3 School of Psychological Sciences, University of Manchester, Oxford Road, Manchester, M13 9PL UK; 4 Manchester Medical School, Manchester University, Manchester, M13 9PL UK; 5 St Mary’s Hospital, Manchester University Hospitals NHS Foundation Trust, Manchester Centre for Genomic Medicine, Manchester, UK; 6 Cardiff University School of Medicine, Institute of Medical Genetics, University Hospital of Wales, Cardiff, UK

**Keywords:** Medical ethics, Health care

## Introduction

Over the past several years, clinicians, patients and families in the UK have witnessed a dramatic change in the way genetic testing is performed, with the introduction of whole genome sequencing (WGS) and the NHS Genomic Test Directory in 2018 [1, 2]. In addition to WGS becoming more accessible, this has increased the chances of receiving a genetic diagnosis, established the reproductive implications of germline genetic variants and, in some patients, guided management [3]. The use of a hybrid (clinical and research) consent form may also open up opportunities to join research studies and clinical trials to more patients [4].

A new version of the consent form (Record of discussion form version 4.03) was introduced in 2021 by the NHS Genomic Medicine Service. This allows the implementation of WSG in mainstream clinical practice, whilst providing patients with the opportunity to donate data and a remainder sample to the National Genomics Research Library. The latter is managed by Genomics England Ltd (GEL), a company set up in 2013 and owned by the (English) Department of Health and Social Care.

The purpose of this article is to explore ways to better support patients, relatives, and clinicians to consider the complex issues of consent in a busy general clinic, so they are better prepared when the result is available. We shall use the core ethical values that underpin consent to highlight some of the issues and, when possible, suggest practical solutions.

## Managing expectations in a busy mainstream clinic

For consent to be valid, the person making the decision should be competent (“have capacity”), have the appropriate information and understand it, and should make their decision voluntarily [4, 5].

The complexity of WGS, the range of possible incidental findings, the inevitable uncertainties, and the often limited understanding about genomics by patients, their family, and sometimes by mainstream clinicians can make informed consent difficult to achieve [5, 6]. Some have argued that an excess of information can be a deterrent for patients, as it may lead to difficulties in ensuring valid consent and hinder access to potentially valuable investigations and treatments [4, 5]. However, it is important that enough information is provided to bring patients’ and families’ hopes and expectations into a realistic alignment with the likely results of WGS.

Up to half of all patients undergoing WGS receive a pathological variant, although this will depend upon the cluster of symptoms and previous investigations undertaken in that individual or family [3]. Patients and families need to know that some patients may receive a clear diagnosis, but others may not, and yet others may receive unexpected and potentially burdensome findings. It is important that they are aware that a genetic diagnosis does not guarantee access to effective treatment or better care [1]. Furthermore, patients and families may struggle with the impact of results of uncertain significance or unsolicited findings. In states of uncertainty, people may just decide that they have the condition and use the associated medical terms to identify themselves [6].

We wonder if the notion of WGS needs clarification when used in discussions with patients. Although each donated DNA sample may have full genome sequencing, it is important for the clinician to explain that only specific segments of the genome, pertaining to the clinical phenotype, will be analyzed and that most of the genome generated will be stored for research or future diagnostic queries. This focused diagnostic strategy is more likely to yield relevant positive results and can also explain why some patients are left with no definite genetic diagnosis at the time of the analysis [1].

## Maintaining trust in a busy mainstream clinic

The consent needs to be “connected” to each patient’s individual context [4]. For instance, in our Motor Neuron Disease clinic, WGS is usually discussed after the diagnosis is confirmed [7]. A time pause, to ensure sufficient psychosocial support is put in place, is crucial for a trusting and compassionate relationship to be maintained with patients and their families [1–4]. In addition, genomic information carries contemporaneous and future implications for both patients and their relatives and may impact decisions about personal aspects of an individual’s life [8]. This information may be relevant in areas beyond health, including employment, sport, education, criminal justice, and insurance. This emphasizes the need for the consent process to be inclusive and dynamic, so that patients and their relatives recognize this potential for serious implications.

The consent form seems to reassure patients that results from diagnostic or predictive testing will not be shared with insurance companies. Companies already require customers to disclose their family history of certain genetic conditions and families may well imagine that genetic test results can also be used to their disadvantage, even though this is currently only true for those at risk of Huntington’s disease (HD) and only when a policy of >£500,000 is being sought [9]. Furthermore, the current policy lacks the force of legislation so that families may reasonably have some concern that existing information may be used against them in the future.

One aspect of GEL that may need clarification is its corporate nature so that patients understand that it is distinct from the NHS. GEL currently controls the processing of all samples and data donated to the Genomic Research Library. To ensure financial sustainability, there could be a future drive to establish commercial ventures that would utilize these samples and data [8]. It is therefore important to be open about this possibility and reflect on ways to ensure this aspect of the genomic research venture does not conflict with the understanding of the patients and clinicians when the consent forms are signed via NHS clinics.

## The further blurring of clinical care and research

Translational genomics may blur the boundaries between clinical practice and research; this may be warranted because the ultimate aim of both clinical care and research is to improve the diagnosis and to offer personalized treatment to patients [1, 4, 5]. We have identified some clear benefits for this hybrid approach in our clinics. For instance, traveling to clinics is time-consuming and expensive for many patients; addressing their clinical needs while offering the opportunity of taking part in research can be cost-effective, timely, and may enhance trust and engagement from diverse communities. Furthermore, multidisciplinary clinics combining research and clinical follow-up have been popular for other conditions such as HD. However, we wonder if the hybrid structure of the consent form should be simplified. Conversations in clinic usually focus on the patient’s diagnosis and consent for clinical testing should be completed before consent for research is broached. Patients are currently asked to opt in or out (YES or NO) of the Genomic Research Library, before being asked to sign the clinical consent form; this may lead some patients to assume that diagnostic testing is conditional upon their joining the research library.

Clinicians should emphasize that declining to participate in research will have no bearing on patients’ clinical care or the chances, in the short to medium term, of receiving a diagnosis. The longer-term chance of a diagnosis may be greater if patients participate in research, but this may come at the cost of receiving information about incidental burdensome findings and generating information about the disease risks faced by other members of their family.

## Conclusion

The commitment of the UK to offer free and comprehensive genomic testing to all patients is clearly praiseworthy, but communication around consent in busy mainstream clinics can be problematic because of what, how, and when information is shared. Patients are often under stress at the time of consultation and may not appreciate the nuances of the consent document.

We acknowledge that obtaining fully informed consent for whole genome sequencing will always be a challenge since one cannot predict all the possible diagnoses or the incidental findings generated. However, we suggest that a time pause is allowed for patients to read the consent form and discuss its substance with their families before making a decision. It may be necessary to remain in contact over time to reaffirm that consent is still forthcoming, especially when novel applications of their genetic information become available.

There is also a clear need for a specific support framework in a busy general clinic where background knowledge about genomic medicine is limited. This could take many forms, including additional training for clinicians, service specifications for mainstream clinics to support additional roles, and extra time being allocated to consultations. When a genomic test is first arranged, it should be clear who would be responsible for the result and who should support patients if they come to be given results of uncertain significance or incidental findings. Practitioners should not wait until the result has been generated before deciding how to return it to the patient. Ultimately, the aim is for patients to feel empowered and to be clear about the meaning of their results in practical terms, including any limitations.

We also believe the current consent document would benefit from a clear demarcation between consent for diagnostic testing and consent for research and that patients should be offered the option to consent for either or both. Finally, we propose that a governance framework should be introduced to protect the interests of everyone involved, including patients, healthcare professionals and private companies, to allow the effective and successful progression of this important field.
